# Supplementary material for: A mitochondrial megachannel resides in monomeric F1FO ATP synthase
Source: Nat Commun. 2019 Dec 20;10:5823. doi: 10.1038/s41467-019-13766-2 (PMC6925261; doi:10.1038/s41467-019-13766-2)
Supplement: Supplementary file 2 — Supplementary Information [file 41467_2019_13766_MOESM2_ESM.pdf]

Supplementary Information for

A mitochondrial megachannel resides in monomeric F_1F_O ATP synthase

Nelli Mnatsakanyan[1], Marc C. Llaguno[2], Youshan Yang[3], Yangyang Yan[3], Joachim Weber[4], Fred J. Sigworth[3], Elizabeth A. Jonas[1]

[1] Section of Endocrinology, Department of Internal Medicine, Yale University, New Haven, CT, USA

[2]Center for Cellular and Molecular Imaging, Yale University, New Haven, CT, USA

[3]Department of Cellular and Molecular Physiology, Yale University, New Haven, CT, USA

[4]Department of Chemistry and Biochemistry, Texas Tech University, Lubbock, TX, USA

Correspondence and requests for materials should be addressed to E.A.J. (email: elizabeth.jonas@yale.edu) or to N.M. (email: nelli.mnatsakanyan@yale.edu)

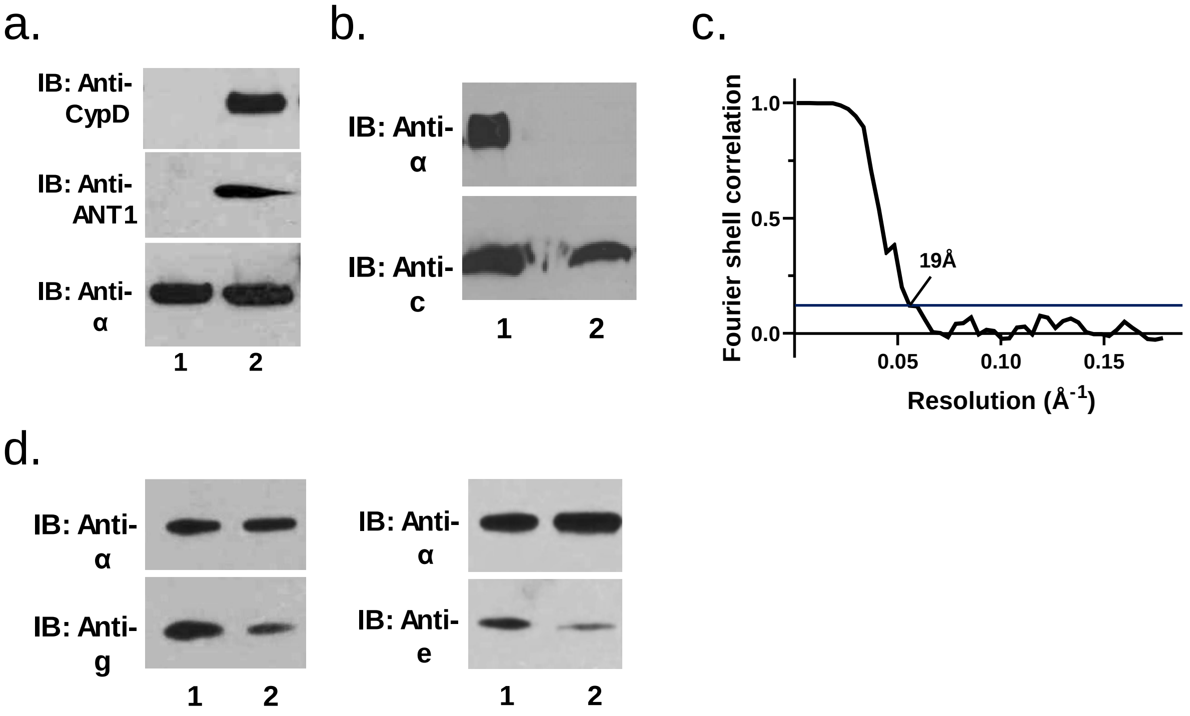

Supplementary Figure 1. a, Immunoblot analysis of ATP synthase. The purified protein (sample 1) and porcine heart mitochondrial lysate (sample 2) were tested for the presence of adenine nucleotide translocator 1 (ANT1) and cyclophilin D (CypD). ATP synthase α-subunit was used as a loading control. The presence of ANT1 and CypD was confirmed in mitochondrial lysate but not in purified ATP synthase fraction after SEC. **b,** Immunoblot analysis of F_1 depleted GUVs. Sodium bromide (3 M) was used to treat ATP synthase reconstituted GUVs. The samples before (sample 1) and after sodium bromide treatment (sample 2) were analyzed. The complete absence of ATP synthase F_1 α-subunit was noted in sample 2. ATP synthase c-subunit was used as a loading control. **c,** Fourier shell correlation (FSC) curve for the 3D reconstruction map of ATP synthase monomer indicates a resolution of ~19 Å at FSC = 0.143. **d,** Porcine heart mitochondrial lysate (sample 1) and purified ATP synthase (sample 2) were tested for the presence of ATP synthase subunits g and e and residual amounts of these proteins were detected. ATP synthase α-subunit was used as a loading control. The blot is a representative of three independent runs. The source data underlying panels **a**, **b**, **d** are provided as a Source Data file.

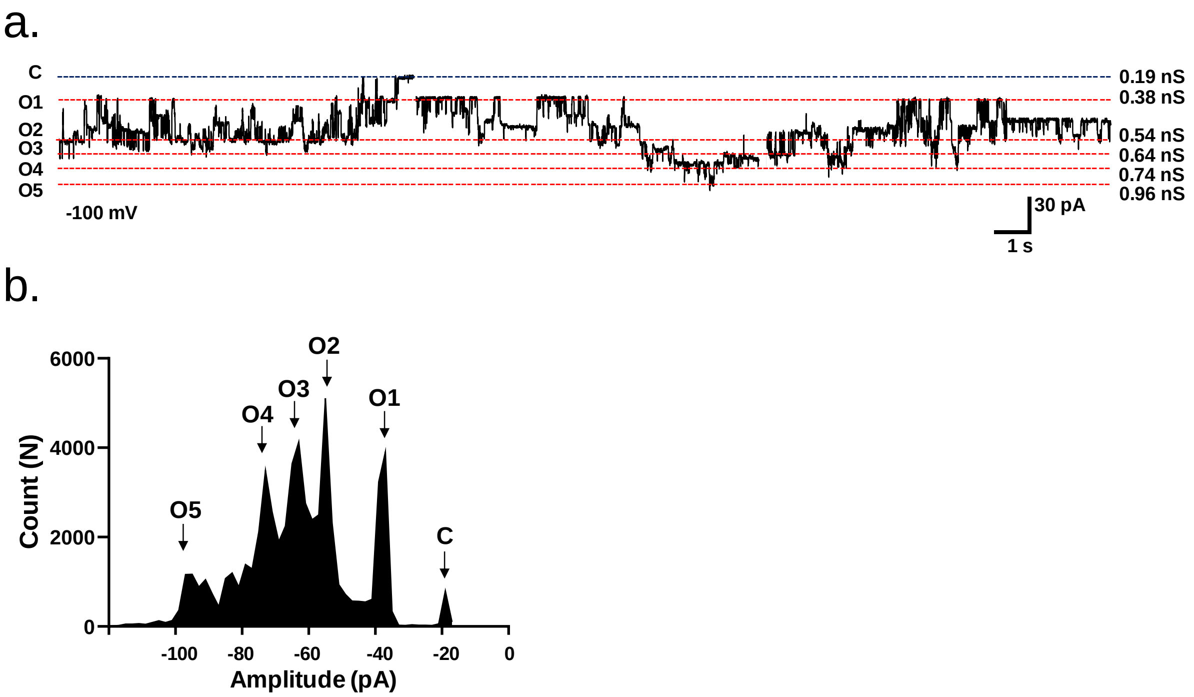

Supplementary Figure 2. Representative patch-clamp recording of an F_1F_O ATP synthase reconstituted liposome. **a,** The channel is open in its multi-conductance mode. The closed (C) and open (O1-O5) states represented by peaks on the amplitude histogram are indicated. **b,** Amplitude histogram representing closed and open states shown in **a**.

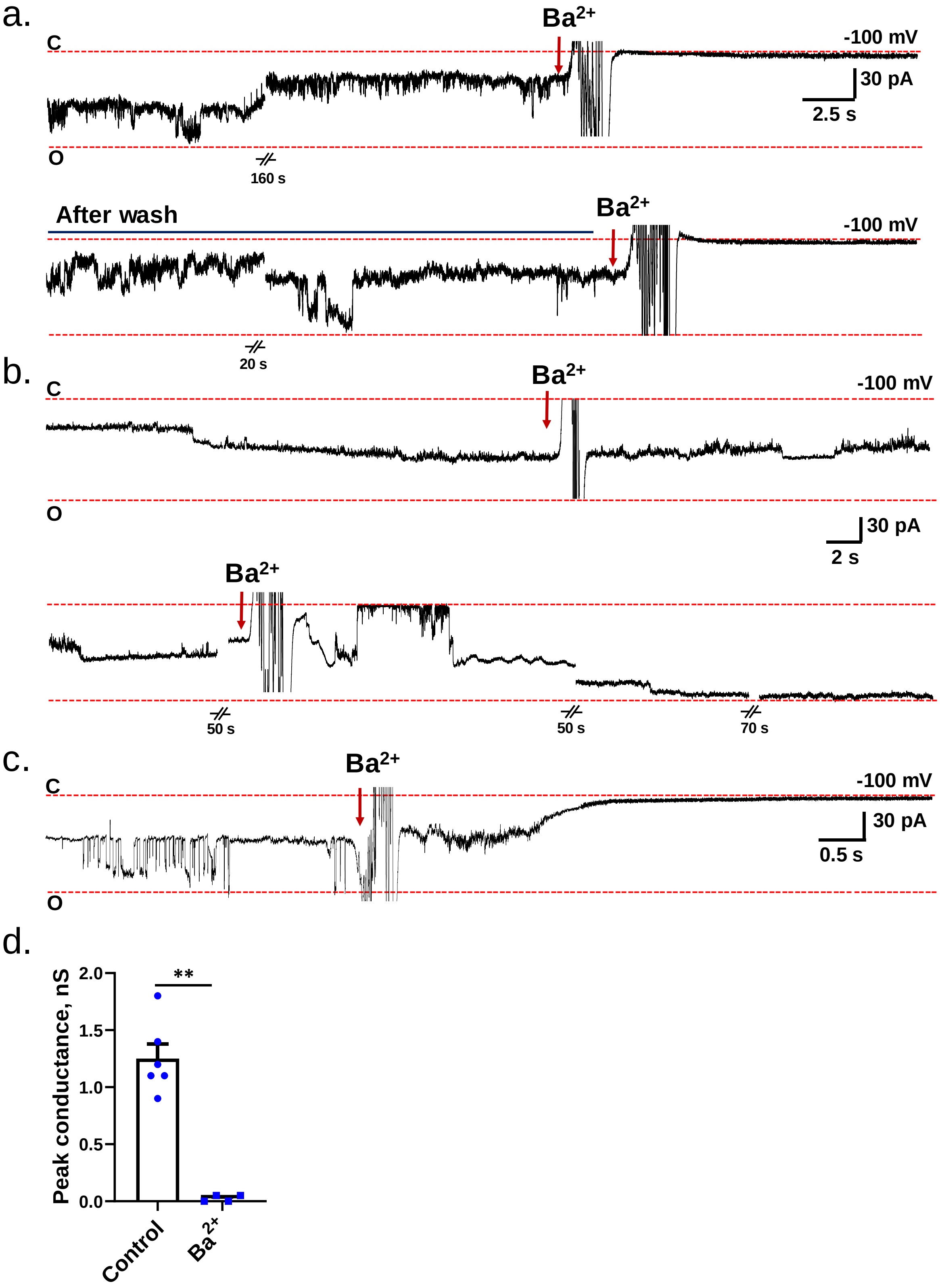
a.
Ba2+
C
-100 mV
30 pA
2.5 s
O
160 s
After wash
Ba2+
-100 mV
20 s
b.
Ba2+
C
-100 mV
O
30 pA
2 s
Ba2+
50 s
50 s
70 s
c.
Ba2+
C
-100 mV
30 pA
0.5 s
O
d.
Peak conductance, nS
2.0
1.5
1.0
0.5
0.0
**
Control
Ba2+

Supplementary Figure 3. Ba^{2+}-sensitive channel inhibition is reversible and F_1-dependent. a. Representative patch-clamp recording of F_1F_O ATP synthase reconstituted liposomes showing reversible closing of channel with Ba^{2+} (1 mM), n=3. Channel reopens after washing the patch with the recording buffer for ~3 minutes and closes again after subsequent addition of barium chloride (1 mM). **b,** Representative patch-clamp recording of F_1-stripped ATP synthase reconstituted liposome after treatment with sodium bromide, n=3 (see Supplementary Figure 1c for western blot analysis). Ba^{2+} (4 mM), which was added in two subsequent additions during continuous recording, failed to close the channel. C, indicates the closed state, O indicates the open state of the channel. **c,** Representative patch-clamp recording of F_1F_O ATP synthase reconstituted in GUVs with the lipid composition of SUVs used in cryo-EM studies. Recording shows closing of the channel with Ba^{2+} (1 mM). **d,** Group data for the peak conductance of ATP synthase channel activity for GUVs such as shown in **c**, before and after addition of Ba^{2+}, n=6 for control, n=4 for Ba^{2+}. Only paired recordings before and after the addition of Ba^{2+} were used for statistical analysis, **$P<0.0055$, paired t test, error bars refer to SEM. The source data underlying panel **d** are provided as a Source Data file.
