# Supplementary material for: A mitochondrial megachannel resides in monomeric F1FO ATP synthase
Source: Nat Commun. 2019 Dec 20;10:5823. doi: 10.1038/s41467-019-13766-2 (PMC6925261; doi:10.1038/s41467-019-13766-2)
Supplement: Supplementary file 3 — Description of Additional Supplementary Files [file 41467_2019_13766_MOESM3_ESM.pdf]

Description of Additional Supplementary Files

File Name: Supplementary Data 1

Description: **Identification and quantification of proteins by LC-MS/MS analysis.** Scaffold software was used for quantitative analysis of proteins to calculate the exponentially modified protein abundance index (emPAI). Proteins were listed based on their emPAI values. Normal and decoy database searches were run, with the confidence level set to 95% ($p<0.05$). Protein identifications were accepted if they could be established at greater than 99.0% probability and contained at least 2 identified peptides. Small hydrophobic membrane proteins cannot be well identified by LC-MS/MS. ATP synthase c-subunit was detected only after digestion with chymotrypsin, but not with trypsin. Only one peptide was identified for subunit A6L and therefore it was excluded from this dataset. ATP synthase membrane subunits e and g were not identified by LC-MS/MS, however their partial presence was detected by using antibodies against these subunits in immunoblots (Supplementary Fig. 1d).
