# Supplementary material for: A mitochondrial megachannel resides in monomeric F1FO ATP synthase
Source: Nat Commun. 2019 Dec 20;10:5823. doi: 10.1038/s41467-019-13766-2 (PMC6925261; doi:10.1038/s41467-019-13766-2)
Supplement: Supplementary file 5 — Reporting Summary [file 41467_2019_13766_MOESM5_ESM.pdf]

nature research

Corresponding author(s): Elizabeth Jonas, Nelli Mnatsakanyan
NCOMMS-19-12463

Last updated by author(s): Nov 13, 2019

Reporting Summary

Nature Research wishes to improve the reproducibility of the work that we publish. This form provides structure for consistency and transparency in reporting. For further information on Nature Research policies, see Authors & Referees and the Editorial Policy Checklist.

Statistics

For all statistical analyses, confirm that the following items are present in the figure legend, table legend, main text, or Methods section.

n/a	Confirmed	
☐	☒	The exact sample size (n) for each experimental group/condition, given as a discrete number and unit of measurement
☐	☒	A statement on whether measurements were taken from distinct samples or whether the same sample was measured repeatedly
☐	☒	The statistical test(s) used AND whether they are one- or two-sided<br>*Only common tests should be described solely by name; describe more complex techniques in the Methods section.*
☐	☒	A description of all covariates tested
☒	☐	A description of any assumptions or corrections, such as tests of normality and adjustment for multiple comparisons
☐	☒	A full description of the statistical parameters including central tendency (e.g. means) or other basic estimates (e.g. regression coefficient) AND variation (e.g. standard deviation) or associated estimates of uncertainty (e.g. confidence intervals)
☐	☒	For null hypothesis testing, the test statistic (e.g. F, t, r) with confidence intervals, effect sizes, degrees of freedom and P value noted<br>*Give P values as exact values whenever suitable.*
☐	☒	For Bayesian analysis, information on the choice of priors and Markov chain Monte Carlo settings
☐	☒	For hierarchical and complex designs, identification of the appropriate level for tests and full reporting of outcomes
☒	☐	Estimates of effect sizes (e.g. Cohen's d, Pearson's r), indicating how they were calculated

Our web collection on statistics for biologists contains articles on many of the points above.

Software and code

Policy information about availability of computer code

Data collection	pCLAMP-10 software was used for patch-clamp electrophysiology data acquisition (Molecular devices).
Data analysis	Patch-clamp electrophysiology data were analyzed by using pClamp 10.0 software (Molecular devices). Relion-1.3 software was used for cryo-EM data analyzes. LC-MS/MS data were analyzed by using Byonic Software (Protein Metrics, San Carlos, CA USA; version PMI-Byonic-Demo:v2.15.7). Cryo-EM models were visualized by using UCSF Chimera.

For manuscripts utilizing custom algorithms or software that are central to the research but not yet described in published literature, software must be made available to editors/reviewers. We strongly encourage code deposition in a community repository (e.g. GitHub). See the Nature Research guidelines for submitting code & software for further information.

Data

Policy information about availability of data

All manuscripts must include a data availability statement. This statement should provide the following information, where applicable:

- Accession codes, unique identifiers, or web links for publicly available datasets
- A list of figures that have associated raw data
- A description of any restrictions on data availability

ATP synthase cryo-EM models from the Figure 2 have been deposited into the Electron Microscopy Data bank (EMDB). LC-MS/MS data have been deposited in the ProteomeXchange Consortium via the PRIDE partner repository. Dataset identifier numbers for both of the submissions have been added to the manuscript.

Field-specific reporting

Please select the one below that is the best fit for your research. If you are not sure, read the appropriate sections before making your selection.

☒ Life sciences ☐ Behavioural & social sciences ☐ Ecological, evolutionary & environmental sciences

For a reference copy of the document with all sections, see nature.com/documents/nr-reporting-summary-flat.pdf

Life sciences study design

All studies must disclose on these points even when the disclosure is negative.

Sample size	For all experiments the number of samples/groups was estimated with power analysis, by keeping alpha at 0.05, power 80%. There are several power analysis calculators that we used for sample size calculations, including the commonly used software located in the following websites: http://biostat.mc.vanderbilt.edu/wiki/Main/PowerSampleSize; http://www.math.yorku.ca/SCS/Online/power/
Data exclusions	No data were excluded.
Replication	We verified the reproducibility of the experimental findings by setting up the difference in the value between the two results which was greater than 95% probability of occurrence.
Randomization	Paired recordings were used before and after treatment for data analysis. Therefore no randomization was used for these studies.
Blinding	Paired recordings were used before and after treatment for data analysis. Therefore no blinding was used for these studies.

Reporting for specific materials, systems and methods

We require information from authors about some types of materials, experimental systems and methods used in many studies. Here, indicate whether each material, system or method listed is relevant to your study. If you are not sure if a list item applies to your research, read the appropriate section before selecting a response.

Materials & experimental systems

n/a	Involved in the study	
☐	☒	Antibodies
☒	☐	Eukaryotic cell lines
☒	☐	Palaeontology
☒	☐	Animals and other organisms
☒	☐	Human research participants
☒	☐	Clinical data

Methods

n/a	Involved in the study	
☒	☐	ChIP-seq
☒	☐	Flow cytometry
☒	☐	MRI-based neuroimaging

Antibodies

Antibodies used	The following antibodies were used in the study: ATP synthase alpha-subunit (Anti-ATP5A ab14748, Abcam); c-subunits (Anti-ATP5G1/G2/G3, ab180149, Abcam), ANT1 (32484, Sabbiotech); CypD (TA302580, Origene). e-subunit (ab122241, Abcam), g-subunit (ab126181, Abcam).
Validation	Mouse monoclonal ATP5A antibody (ab14748) was validated in WB, IP, IHC, ICC, Flow Cyt, ICC/IF and tested in Mouse, Rat, Cow, Human, Pig, Caenorhabditis elegans, Drosophila melanogaster, Monkey. Rabbit monoclonal Anti-ATP5G1/G2/G3 antibody (ab180149) was validated in WB, IP, IHC and tested in Mouse, Rat, Human, Pig. Rabbit polyclonal ANT1 antibody (32484) was validated in WB, IHC and tested in Mouse, Rat, Human, Pig. Goat polyclonal antibody (TA302580) was validated in WB and tested in Human and Pig. e-subunit (ab122241, Abcam) and g-subunit (ab126181, Abcam) antibodies were validated in WB.
